# Bat Employs a Conserved MDA5 Gene to Trigger Antiviral Innate Immune Responses

**DOI:** 10.3389/fimmu.2022.904481

**Published:** 2022-05-23

**Authors:** Jie Wang, Zhenyu Lin, Qiuju Liu, Feiyu Fu, Zhaofei Wang, Jingjiao Ma, Hengan Wang, Yaxian Yan, Yuqiang Cheng, Jianhe Sun

**Affiliations:** Shanghai Key Laboratory of Veterinary Biotechnology, Key Laboratory of Urban Agriculture (South), Ministry of Agriculture, School of Agriculture and Biology, Shanghai Jiao Tong University, Shanghai, China

**Keywords:** BAT, MDA5, antiviral innate immunity, virus, IFNβ

## Abstract

Bats are important hosts for various zoonotic viral diseases. However, they rarely show signs of disease infection with such viruses. As the first line for virus control, the innate immune system of bats attracted our full attention. In this study, the *Tadarida brasiliensis* MDA5 gene (batMDA5), a major sensor for anti-RNA viral infection, was first cloned, and its biological functions in antiviral innate immunity were identified. Bioinformatics analysis shows that the amino acid sequence of batMDA5 is poorly conserved among species, and it is evolutionarily closer to humans. The mRNA of batMDA5 was significantly upregulated in Newcastle disease virus (NDV), avian influenza virus (AIV), and vesicular stomatitis virus (VSV)-infected bat TB 1 Lu cells. Overexpression of batMDA5 could activate IFNβ and inhibit vesicular stomatitis virus (VSV-GFP) replication in TB 1 Lu cells, while knockdown of batMDA5 yielded the opposite result. In addition, we found that the CARD domain was essential for MDA5 to activate IFNβ by constructing MDA5 domain mutant plasmids. These results indicated that bat employs a conserved MDA5 gene to trigger anti-RNA virus innate immune response. This study helps understand the biological role of MDA5 in innate immunity during evolution.

## Introduction

Bats are one of the most abundant and geographically distributed vertebrates on earth. There are more than 1,300 species of bats, and they are considered to be the second most ancient mammal species after rodents (1, 2). Bats are hosts of zoonotic viruses such as rabies, Hendra and Nipah, which are highly pathogenic to humans. In addition, bats are increasingly recognized as the reservior host of coronaviruses, such as severe acute respiratory syndrome (SARS) and the Middle East respiratory syndrome (MERS), and the 2019 outbreak of coronaviruses (CoVID-19), which have caused deadly diseases in humans and animals (3–6). However, bats rarely display signs of disease upon viral infection.

The host immune system plays an important role in preventing viral infection and replication. Studies have found that both innate immunity and adaptive immunity exist in the immune system of bats (7). Innate immunity is the first line of defense against virus invasion. Viruses or pathogen-associated molecular patterns (PAMPS) can be recognized by the host pattern recognition receptors (PRRs) and induce antiviral responses (8). The pattern recognition receptors in the host mainly include toll-like receptors (TLRs), retinoic acid-inducible gene I (RIG-I)-like receptors (RLRs), and nucleotide-binding oligomerization domain (NOD)-like receptors (NLRs) (9). Studying flying fox and a few other bat species have identified PRRs known in humans are conserved in bats, including TLRs and RLRs. Viruses, bacteria, fungi, and parasitic pathogens invade the host and can be recognized by TLRs and induce the host’s innate immune response (10). There are 10 TLRs (TLR1-10) in humans (11). The black flying fox and the fruit bat have TLR1-10 and TLR-13, of which TLR-13 is absent in humans and most mammals (12). The RLRs family mainly recognizes and binds viral RNA nucleic acids in the cytoplasm to activate the downstream antiviral immune response (13). Transcriptome analysis revealed that RLRs family members of retinoic acid-inducible gene I (RIG-I), melanoma differentiation-associated gene 5 (MDA5), and laboratory of genetics and physiology 2 (LGP2) existed in the black flying fox and the bat RLRs were similar in structure and expression to human (2, 14).

RIG-I and MDA5 share many structural similarities, and both contain two caspase activation and recruitment domains (CARD), a central DEAD helicase domain (ATP), and a C-terminal repressor domain (CTD) (15, 16). The ATP domain can utilize ATP hydrolysis to unwind and bind viral RNA. The CTD domain has an auto-inhibitory function, which can only be released after binding to RNA virus nucleic acid. Its existence effectively prevention the continuous activation of host immune signals. The CARD domain can transmit signals to downstream adapters, the mitochondrial antiviral signaling protein (MAVS), to induce the production of type I interferons or cytokines (17, 18). The study found that stimulation of bat kidney cells with synthetic dsRNA (ploy: IC) significantly upregulated the expression of three RLRs, suggesting that bat RLRs function similarly to humans (14). RLRs can activate downstream interferon signaling pathways to exert antiviral effects after RNA virus infection. Previous studies have shown that the bat type I interferon gene IFNα is constitutively expressed in unstimulated bats cells and tissues. However, infection with virus or stimulation of ploy: IC did not affect its expression (19). Therefore, the researchers hypothesized that bats control viral replication in the early stages of the immune response through antiviral mechanisms (2).

The coronaviruses (CoVID-19) outbreak has brought serious disasters worldwide (20). However, bats have no signs of disease as the natural host of many coronaviruses, including CoVID-19 (21, 22). Therefore, bats’ unique innate immune system has attracted great attention. *Tadarida brasiliensis* is one of the most widespread mammalian species in the Western Hemisphere, inhabiting a wide range of urban and wild environments. Researchers have isolated and identified a variety of viruses from the bats that are likely to transmit spontaneously the viruses to humans and other animals due to the specificity of their migration and habitat (23, 24). In this study, we cloned *Tadarida brasiliensis* MDA5 for the first time, analyzed its function, explored the differences in bats’ innate immunity, and explained the coexistence of bats with viruses. We found that the amino acid sequence of batMDA5 is poorly conserved among species, and RNA viruses such as NDV, VSV-GFP, and AIV infection *Tadarida brasiliensis* TB 1 Lu cells significantly upregulate the expression of batMDA5. Overexpression of batMDA5 noticeably activates the expression of IFNβ, MX1, and OAS1 and inhibits the VSV-GFP virus replication, while knockdown of batMDA5 yields the opposite results. At the same time, we also found that the CARD domain is essential for the activation of IFNβ by batMDA5. Therefore, we found that bat MDA5 can activate IFNβ to inhibit virus replication in this study.

## Materials and Methods

### Cell Culture and Virus

Chicken embryonic fibroblast cell line DF1, human 293T cells, and bat TB1 Lu cells were obtained from ATCC and cultured in DMEM supplemented with 10% FBS and cells were incubated at 37°C in a 5% CO2 incubator. Newcastle disease virus (NDV-GFP) was a low virulent strain LaSota named NDV-GFP. Avian influenza virus (AIV) were A/Chicken/Shanghai/010/2008 (H9N2) virus (SH010) was isolated from chicken in Shanghai, China, in 2008 and identified as H9N2 avian influenza A virus. The GFP tagged vesicular stomatitis virus (VSV) VSV-GFP were stored in our Laboratory. The viruses were purified, propagated, and stored as described in our previous study (25).

### Cloning and Bioinformatics Analysis of batMDA5

Based on the Molossus molossus MDA5 sequence (NW_023425346.1) obtained from the National Center for Biotechnology Information (NCBI), the primers batMDA5-F and batMDA5-R (**Supplementary Table 1**) were designed and used to amplify batMDA5 cDNA *via* RT-PCR from TB 1 Lu cells. The PCR product was ligated into a pTOPO-Blunt vector (Aidlab, Beijing, China) for sequencing, and the positive colonies were sent to the Beijing Genomics Institute (Beijing, China) for sequencing. The amino acid sequence of batMDA5 was aligned with the other animal MDA5 proteins from mammals such as humans, mice, pigs, goats, and bats; Birds and Reptiles such as chicken, ducks, goose, and fishes such as zebrafish, common carp, and black crap using Clustal W and edited with ESPript 3.0 (http://http://espript.ibcp.fr/ESPript/cgi-bin/ESPript.cgi) as previously described (26). Sequence homology and phylogenetic analysis of the MDA5 amino acid sequences were conducted using DNASTAR. A phylogenetic tree was constructed based on the MDA5 from 30 different species, including mammals, birds, fish, and 10 different bat species. Different domains in the MDA5 amino acid sequences were predicted using the simple modular architecture research tool (SMART) program (http://smart.embl-heidelberg.de/) as previously described (27). Homology modeling for MDA5 was conducted using the online protein-modeling server SwissModel (http://swissmodel.expasy.org/) as previously described (28).

### Plasmid Construction

pcDNA3.1-batMDA5-FLAG plasmids were constructed by inserting full-length batMDA5 into the HindIII, and EcoRI sites pcDNA3.1-FLAG of the expression vector using a ClonExpress II one-step cloning kit (Yeasen, Shanghai, China). The primers used in the PCR are listed in **Supplementary Table 1**. The truncated plasmids of batMDA5, including CARD1 domain, CARD2 domain, DEXDc domain (ATP), HELICc domain (CTD), CARD domain, were constructed using a modified homologous recombination method and the primers listed in **Supplementary Table 1**. The chIFN-β and huIFN-β promoter-luciferase reporter plasmids (pGL-IFN-β-Luc). The DH5α Chemically Competent Cell (Tsingke Biology Technology, Beijing, China) was used for plasmid transformation. The pGL-IFN-β-Luc plasmid was constructed in our previous study (29).

### Cells Transfection

DF1, 293T, and TB 1 Lu cells were seeded in 12-well or 24-well plates (NEST Biotechnology, Wuxi, China) at 5 x 10^5^/mL or 1 x 10^6^/mL. And the plasmid were transfected 500 ng/well in 12-well or 1000 ng/well in 6-well with Nulen PlusTrans™ Transfection Reagent (Nulen, Shanghai, China) according to the manufacturer’s protocol.

### RNA Extraction and qPCR

Cells’ total RNAs were extracted with AG RNAex Pro Reagent (Accurate Biology, Hunan, China). mRNA was reverse‐transcribed to cDNA with reverse transcription kits (Vazyme, Nanjing, China), and the cDNA was analyzed using the SYBR green PCR mix (Vazyme, Nanjing, China) with the Applied Biosystems machine (ABI 7500; Thermo Fisher Scientific). Relative gene expression was analyzed using the 2^−ΔΔCt^ method. The β‐actin was the internal reference when examining the level of genes. The primer sequences for the genes are shown in **Supplementary Table 1**.

### Western Blot Analysis

The cells’ total proteins were extracted by radioimmunoprecipitation assay (Beyotime, Shanghai, China) containing a protease cocktail (Yeasen, Shanghai, China) and phenylmethylsulfonyl fluoride (PMSF) (Yeasen, Shanghai, China). The lysate was centrifuged at 13,000 rpm for 10 min to obtain the supernatant, and a 5 × SDS loading buffer was added before the lysates were boiled for 10 min. The proteins isolated from the cell lysates were separated *via* SDS-PAGE and analyzed using Western blot. The antibody included ant-FLAG (Nulen, Shanghai, China) and β-tubulin overnight at 4°C. The membrane was washed 3 times with tris buffered saline and Tween-20 (TBST) (Sangon Biotech Co., Ltd, Shanghai, China). Then, the secondary antibody was added for 1 h incubation at 4°C shaker Images were obtained using the Tanon 5200 imaging system (Tanon, Shanghai, China).

### Luciferase Reporter Assay

The DF-1, 293T, and TB 1 Lu cells were plated in 24-well plates and were transiently transfected with the reporter plasmid pGL-chIFN-β-Luc or pGL-HuIFN-β-Luc (120 ng/well) and the control Renilla luciferase (pRL-TK, 60 ng/well). According to the manufacturer’s instructions, the cells were lysed 24 hours after transfection, and luciferase activity was detected using a Dual-Luciferase Reporter Assay System kit (Promega, Madison, WI). Renilla luciferase activity was used for normalization. All reporter assays were repeated at least three times.

### Statistical Analysis

Results are expressed as the mean ± SD. GraphPad Prism 8.0 was utilized to graph the results. Data were analyzed by using a two-tailed independent the Student’s *t*-test. *P* < 0.05 was considered statistically significant, and *P* < 0.01 was considered highly statistically significant (*P < 0.05; **P < 0.01).

## Results

### Conservation Analysis of batMDA5

**Figure 1:**
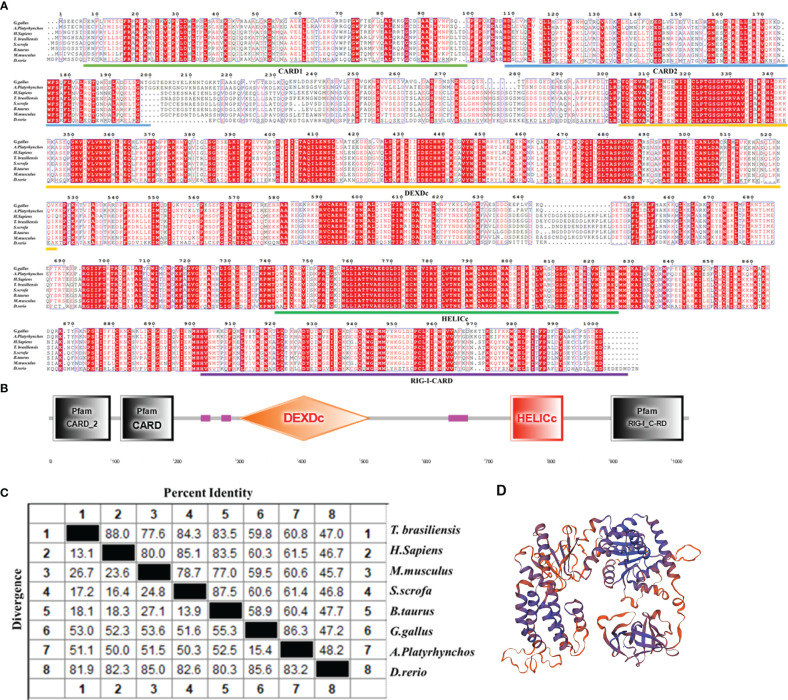
Conservation analysis of batMDA5. **(A)** The alignment of the deduced amino acid sequence of Tadarida brasiliensis MDA5 with other animal MDA5 proteins from the chicken, duck, human, mouse, pig, and fish was performed using the Clustal X and edited with ESPript 3.0; **(B)** Protein domains of batMDA5 predicted by SMART; **(C)** The amino acid sequence homology of different animals; **(D)** Three-dimensional structure of batMDA5 predicted by SwissModel.

To clarify the biological function of bat MDA5 in antiviral innate immunity, we amplified Tadarida brasiliensis MDA5 using the Tadarida brasiliensis 1 lung cell line (TB 1 Lu) cDNA. The ORF of batMDA5 contains 3084 bp and encodes 1027 amino acids residues (**Figure 1A**). After predicting the secondary structure of batMDA5 through the online website SMART (http://smart.embl-heidelberg.de/), it was found that batMDA5 has four typical domains of CARD DEXDc, HELICc, and RIG-I-CARD (**Figure 1B**). Multiple sequence analysis found that batMDA5 is poorly conserved among species, and the similarity with mammals such as humans (NC_000002.12), mice (NC_000068.8), pig (NC_010457.5), and cattle (NC_037329.1) respectively 88.0%, 77.6%, 84.3%, and 83.5%. The similarity with poultry were 59.8% (chicken NC_052538.1), 60.7% (duck NC_051778.1). The lowest similarity to fish was 47.0% (zebrafish NC_007120.7) (**Figure 1C**). Also, there are multiple species of bats. After analyzing the batMDA5 sequences of NCBI, we found that the batMDA5-encoded amino acid sequences size was similar in different bats species, but the similarity is very low (**Figure S1A**). Tadarida brasiliensis has the highest similarity with the common vampire bat, Jamaican fruit-eating bat, pale spear-nosed bat, and Chinese rufous horseshoe bat, respectively 56.3%, 55.2%, 56.9%, 56.3% (**Figure S1B**). Next, we used SwissModel to predict the protein structure of batMDA5 (**Figure 1D**).

### Phylogenetic Tree Analyses of batMDA5

**Figure 2:**
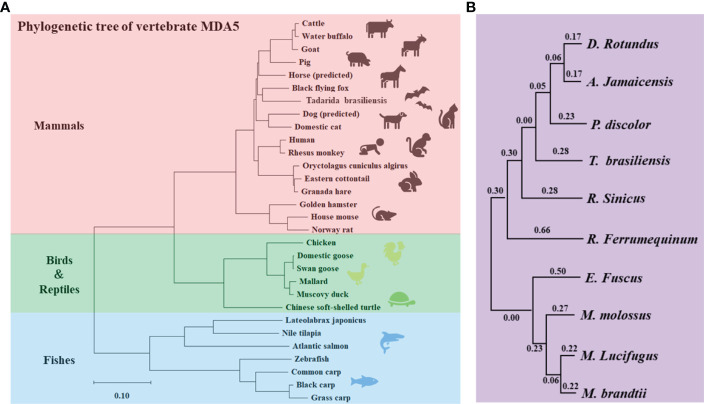
Phylogenetic Tree Analyses of batMDA5. **(A)** Phylogenetic tree of the deduced amino acid sequence of batMDA5 and other animal MDA5 proteins; **(B)** Phylogenetic tree of the deduced amino acid sequence of MDA5 among different bat species.

As an RNA virus sensor, MDA5 plays an important role in the process of antiviral innate immunity. In this study, we found that the batMDA5 has the highest similarity with humans, only 80%. And the similarity within species, Tadarida brasiliensis batMDA5 has the highest similarity with the common vampire bat, only 56.3%. This indicates that batMDA5 is less conserved among species. We performed a phylogenetic tree analysis of MDA5 in mammals, birds, reptiles, and fish to elucidate its molecular function further. As mammals, bats are more closely related to humans, pigs, dogs, and horses in evolution, and farther related to birds, fish, and reptiles (**Figure 2A**). Similarly, after analyzing the batMDA5 phylogenetic tree between bats, we found that batMDA5 also has a complex evolutionary relationship among bats, Tadarida brasiliensis with a common vampire bat, Jamaican fruit-eating bat, pale spear-nosed bat, and Chinese rufous horseshoe bat belong to the same branch in evolution (**Figure 2B**).

### Upregulation of batMDA5 Expression in Response to RNA Viral Infection

**Figure 3:**
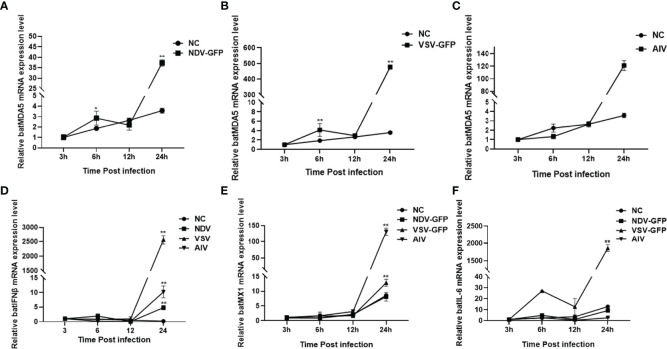
Upregulation of batMDA5 expression in response to RNA viral infection. **(A)** RT-qPCR was used to detect the expression level of batMDA5 in TB 1 Lu cells infection with NDV at 1.0 MOI. **(B)** RT-qPCR was used to detect the expression level of batMDA5 in TB 1 Lu cells infected with VSV at 1.0 MOI. **(C)** RT-qPCR was used to detect the expression level of batMDA5 in TB 1 Lu cells infection with AIV at 1.0 MOI. **(D)** RT-qPCR was used to detect the expression level of batIFNβ in TB 1 Lu cells infection with NDV, VSV, and AIV at 1.0 MOI. **(E)** RT-qPCR was used to detect the expression level of batMX1 in TB 1 Lu cells infection with NDV, VSV, and AIV at 1.0 MOI. **(F)** RT-qPCR was used to detect the expression level of batIL-6 in TB 1 Lu cells infection with NDV, VSV, and AIV at 1 MOI. Data are expressed as the means ± SD of three independent experiments. *P < 0.05; **P < 0.01.

Bats are natural hosts of many RNA viruses. Previous studies have found that Newcastle disease virus (NDV-GFP), vesicular stomatitis virus (VSV-GFP), and avian influenza virus (AIV) can infect bats. And NDV, AIV and VSV infection significantly upregulate mammalian or avian MDA5 expression levels. However, the effect of RNA viruses on bat MDA5 expression remains unclear. Therefore, in this study, we infected the TB 1 Lu cell line with RNA viruses such as NDV-GFP, VSV-GFP, and AIV. We detected the expression levels of batMDA5 and immune-related genes at infected with the virus for 3, 6, 12, and 24 h, respectively. The results showed that infection of TB 1 Lu cells with the above three RNA viruses could significantly upregulate the expression of batMDA5 (**Figures 3A–C**). Moreover, they also significantly upregulate the expression of immune-related genes such as batIFNβ, batMX1, and IL-6 mRNA (**Figures 3D–F**).

### Overexpression of batMDA5 Promoted Bats Antiviral Innate Immunity During VSV-GFP Infection

**Figure 4:**
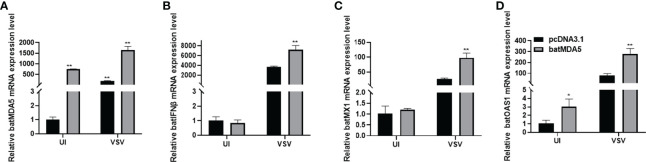
Overexpression of batMDA5 promoted bats’ antiviral innate immunity during VSV-GFP infection. **(A)** RT-qPCR was used to detect the overexpression efficiency of batMDA5 in TB1 Lu cells transfected with 500 ng/well of pcDNA3.1 or pcDNA3.1-batMDA5 uninfected (UI) or infected with VSV-GFP at 1.0 MOI. **(B–D)** RT-qPCR was used to detect the overexpression level of IFNβ, MX1, and OAS1 in TB 1 Lu cells after overexpression of pcDNA3.1-batMDA5, uninfected (UI) or infected with VSV-GFP at 1.0 MOI. Data are expressed as the means ± SD of three independent experiments. *P < 0.05; **P < 0.01.

To further explore the role of MDA5 in innate antiviral immunity, we transfection batMDA5 plasmids in the TB 1 Lu cells, collected the cells after infecting VSV-GFP for 24 h and detected the immune-related gene expression. It was found that overexpression of batMDA5 significantly promotes the expression of batIFNβ and interferon-stimulated related genes batMX1 and batOAS1 after infection with VSV-GFP (**Figures 4A–D**), suggesting that batMDA5 can sense RNA virus invasion and promote batIFNβ expression.

### Overexpression of batMDA5 Inhibits Vesicular Stomatitis Virus (VSV-GFP) Replication

**Figure 5:**
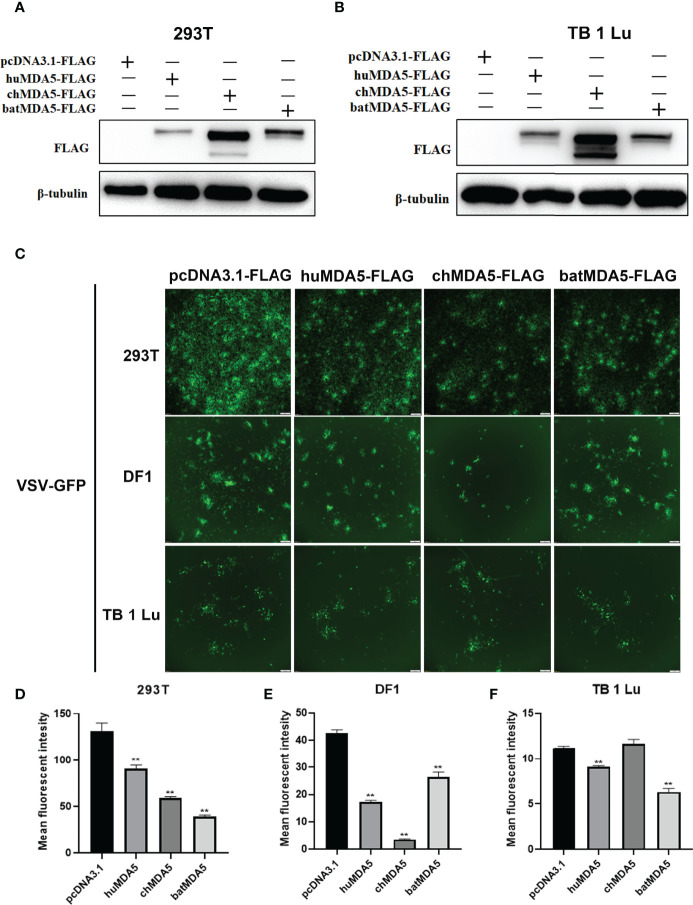
Overexpression of batMDA5 inhibits vesicular stomatitis virus (VSV-GFP) replication. **(A, B)** Western bolting analysis of the expression of pcDNA3.1-huMDA5-flag or pcDNA3.1-chMDA5-flag or pcDNA3.1-batMDA5-flag or pcDNA3.1-flag in 293T and TB 1 Lu cells after overexpression of pcDNA3.1-huMDA5-flag or pcDNA3.1-chMDA5-flag or pcDNA3.1-batMDA5-flag or pcDNA3.1-flag infected with VSV-GFP at 1.0 MOI for 12 h. **(C)** Viral fluorescence in 293T, DF1 and TB 1 Lu cells after overexpression of pcDNA3.1-huMDA5-flag or pcDNA3.1-chMDA5-flag or pcDNA3.1-batMDA5-flag or pcDNA3.1-flag infected with VSV-GFP at 1.0 MOI for 12 h. **(D–F)** VSV-GFP mean fluorescent intesity in 293T, DF1 and TB 1 Lu cells after overexpression of pcDNA3.1-huMDA5-flag or pcDNA3.1-chMDA5-flag or pcDNA3.1-batMDA5-flag or pcDNA3.1-flag infected with VSV-GFP at 1.0 MOI for 12 h. Data are expressed as the means ± SD of three independent experiments. **P < 0.01.

Next, we explored the role of batMDA5 in virus replication. After overexpression of huMDA5, chMDA5, and batMDA5 in human 293T cells, chicken DF1 cells, and bat TB 1 Lu cells, VSV-GFP was infected for 12 h and detected the expression of MDA5 in the cells by Western blot (**Figures 5A, B**). The fluorescence intensity of VSV-GFP reflects its replication in cells. Through fluorescence microscope observation, we found that overexpression of batMDA5 in 293T and TB 1 Lu cells obviously inhibits the replication of VSV-GFP, but in DF1, only chMDA5 significantly inhibited the replication of GFP-VSV, huMDA5, and batMDA5 do not affect controlling VSV-GFP replication in DF1 cells (**Figures 5C–F**). The results above indicate that batMDA5 inhibits virus replication by activating the innate immunity of bats.

### Knockdown of batMDA5 Inhibits Bats Antiviral Innate Immunity Promotes the VSV-GFP Replication

**Figure 6:**
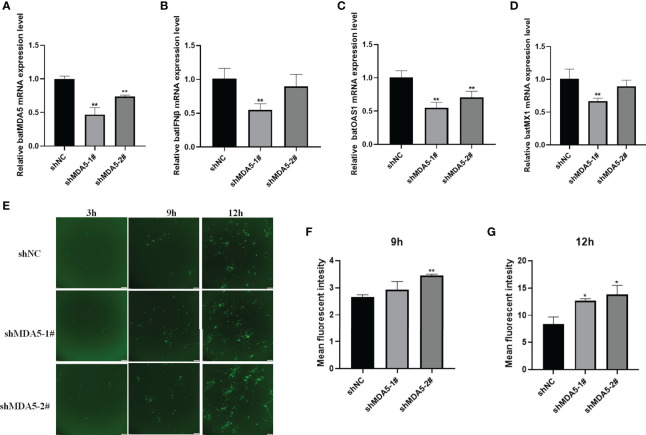
Knockdown of batMDA5 inhibits bats’ antiviral innate immunity and promotes the VSV-GFP replication. **(A)** RT-qPCR was used to detect the Knockdown efficiency of batMDA5 in TB 1 Lu cells transfected with shNC or shMDA5-1# or shMDA5-3#, infected with VSV-GFP at 1 MOI for 12 h **(B–D)** RT-qPCR was used to detect the expression level of IFNβ, MX1 and OAS1 in TB 1 Lu cells after knockdown of batMDA5, infected with VSV-GFP at 1.0 MOI for 12 h **(E)** Viral fluorescence in TB 1 Lu cells, after knockdown of batMDA5, infected with VSV-GFP at 1 MOI for 3 h, 9 h and 12 h **(F, G)** The VSV-GFP mean fluorescent intesity in 2 TB 1 Lu cells after knockdown of batMDA5, infected with VSV-GFP at 1.0 MOI for 9 h and 12 h Data are expressed as the means ± SD of three independent experiments. *P < 0.05; **P < 0.01.

To further prove the effectiveness of batMDA5 on bat antiviral innate immunity, we constructed RNAi plasmids of batMDA5 plasmids (shMDA5-1#, shMDA5-2#, shNC). The shRNA was transferred into TB 1 Lu cells for 24 h and infected with VSV-GFP for 12 h. The expression level of batMDA5 was detected by RT-qPCR, and it was found that both shMDA5-1# and shMDA5-2# could significantly inhibit the expression of batMDA5 (**Figure 6A**). At the same time, after the knockdown of the expression of batMDA5, infection with VSV-GFP significantly inhibits the expression of batIFNβ and interferon-stimulated related genes batMX1 and OAS1 (**Figures 6B–D**). In addition, we also found that knockdown of batMDA5 could promote the VSV-GFP virus replication (**Figures 6E–G**). The above results further demonstrate that batMDA5 can activate the innate immunity of bat and suppress viral replication.

### The batMDA5 Essential Domain for Activation of the IFNβ

**Figure 7:**
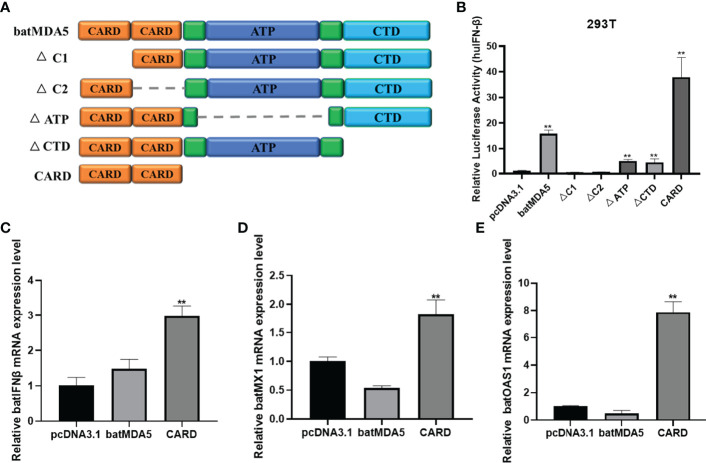
The batMDA5 is an essential adaptor for activation of the IFNβ. **(A)** Schematic structure of batMDA5 mutants. **(B)** 293T cells were co-transfected with luciferase reporter plasmids (pRL-TK and pGL-huIFN-β-Luc) and pcDNA3.1-batMDA5 mutants or pcDNA3.1. After transfection 12 h infection VSV-GFP at 1 MOI, luciferase assays were performed after 24 h of co-transfection. **(C–E)** RT-qPCR was used to detect the expression level of IFNβ, MX1, and OAS1 in TB1 Lu cells after overexpression of batMDA5 or batMDA5-CARD or pcDNA3.1. Data are expressed as the means ± SD of three independent experiments. **P < 0.01.

To further explore the essential functional domains of batMDA5 inactivation of the IFNβ, we constructed batMDA5 mutant plasmids lacking different functional domains of batMDA5 (**Figure 7A**). Their IFNβ activation abilities were assessed with the huIFNβ luciferase report assay in 293T cells. The results found that deletion of the CARD1 and CARD2 domains of batMDA5 loses the function of batMDA5 for activating the huIFNβ promoter. However, the batMDA5-CARD domain strongly activates the huIFNβ promoter (**Figure 7B**). Next, we overexpressed batMDA5 and batMDA5-CARD domains in TB I Lu cells. After overexpression of batMDA5-CARD, batIFNβ and the expression of interferon-stimulated related genes batMX1 and batOAS1 could be significantly activated without virus stimulation. While batMDA5 is in a state of self-inhibition under normal circumstances, overexpression of MDA5 does not activate IFNβ in the absence of viral stimulation (**Figures 7C–E**), suggesting that the CARD domain is critical for batMDA5 to activate batIFNβ.

## Discussion

The host’s innate immune system plays an important antiviral role during viral infection. The host mainly detects the presence of viruses by using recognition receptors, among which MDA5 is an important RNA virus nucleic acid sensor, mainly recognizes and binds to long double-stranded RNA (dsRNA) viruses, which can sense virus invasion and transmit signals to the adapter mitochondrial antiviral Signal protein (MAVS) that promotes the secretion of type I interferon (30). Type I interferons further orchestrate the cellular immune response to viral infection (31). Bats, being mammals, carry many very pathogenic viruses to humans, but they rarely display signs of illness (1). Whether RNA viral recognition sensors such as MDA5 in bat cells can recognize viral nucleic acids and exert antiviral effects remains unknown.

In this study, we cloned *Tadarida brasiliensis* MDA5. We found that batMDA5 and huMDA5 have high homology through sequence analysis and phylogenetic tree analysis. By analyzing the MDA5 of different species of bats, we found that *Tadarida brasiliensis* MDA5 has low homology in different bats. Evolutionarily, batMDA5 is closer to mammals such as humans, pigs, and horses, indicating that MDA5 has homology in species evolution. It has been found that the amino acid sequence of *P. alecto* MDA5 is most closely related to horses in evolution (32). After high-throughput whole-genome sequencing of bats, studies have found that bat-encoding genes and many mammalian genes have a single orthologous copy (33). RIG-I and MDA5 are critical for activating type I interferon expression during viral infection. Phylogenetic analysis suggests that RIG-I and MDA5 come from a common origin, with MDA5 orthologs present in most vertebrates. In contrast, RIG-I orthologs exist only in mammals, ducks, geese, and some specific fish and reptiles (34), suggesting that MDA5 plays an important role across species. So during viral infection, can batMDA5 also recognize RNA viruses to activate antiviral innate immunity?

Mammalian cells or chicken fibroblast DF1 cells infected with RNA virus significantly upregulate the expression level of MDA5 (35–37). In this study, we also found the same results in the TB 1 Lu cells after infecting RNA viruses such as NDV-GFP, AIV, or VSV-GFP upregulated the expression of batMDA5 and innate immunity-related genes batIFNβ, batMX1, and batIL-6, suggesting that the innate immune system of bats also can sense the invasion of the virus and play an antiviral role. It has been found that kidney epithelial cell lines derived from four bat species infected with Encephalomyocarditis virus (EMCV) and Japanese encephalitis virus (JEV) can also significantly upregulate the expression of batMDA5, indicating that a variety of RNA viruses can upregulate batMDA5, and batMDA5 in different bat species appear to be functionally similar (38). Interestingly, after we infected TB 1 Lu cells with three RNA viruses for 12h, the expression levels of batMDA5, batIFNβ, batMX1, and batIL-6 were lower than those of 6h infection. This may be due to the cell cycle or the viral replication cycle. However, the specific reasons still need further research.

Next, we overexpressed batMDA5 in TB 1 Lu cells. The results showed that overexpression of MDA5 could significantly promote the expression of IFNβ and inhibit virus replication after infection with VSV-GFP, indicating that the function of batMDA5 is conserved among species, and batMDA5 can recognize RNA viruses to activate the host’s innate immune response to inhibit virus replication (39). Pteropine Orthoreovirus (PRV) is a zoonotic disease for which bats are its natural host. The study found that the replication ability of PRV in BHK-21 and HEK293T cells was significantly stronger than that in various bat cells. Therefore, the researchers speculate that bats can use their powerful immune systems to suppress virus replication and reduce clinical symptoms (32). To further explore the function of batMDA5, we knocked down its expression and found that it could significantly inhibit the expression of IFNβ and promote virus replication, indicating that batMDA5 could activate IFNβ expression and exert an antiviral effect.

Structurally, batMDA5 also contains four domains, 2CARD, ATP, and CTD (40). In general, the MDA5 CARD domain can interact with the downstream MAVS CARD domain through a series of cascade reactions that ultimately lead to the expression of IFNβ (34, 41). This study found that the amino acid sequence of the CARD region of batMDA5 was very poorly conserved among species. To further explore the structure and function of batMDA5, we constructed batMDA5 plasmids with different functional domains deleted. Deleting CARD1 or CARD2 was found that batMDA5 loses the ability to activate IFNβ. On the contrary, only retaining the CARD domain can strongly activate IFNβ, indicating that the function of batMDA5 is conserved in evolution.

Combining the above studies, we found that batMDA5 is conserved between species in structure and function, suggesting that batMDA5 can activate downstream antiviral immune responses to inhibit viral replication after bat infection with RNA viruses. Nevertheless, how do viruses settle in bats without being cleared by the host’s innate immune system? Relevant studies have shown that several immune-related genes have high expression levels in bats, including RIG-I, MDA5, IFNα, and IRF family, which makes viruses recognized by the immune system and quickly eliminated once they invade bats (42–44). However, a rapid and intense immune response can cause a “cytokine storm” that damages host tissues and organs and leads to death (45). To avoid this severe consequence, bats evolved to activate genes for inflammation or IFNs, which either lost or altered their function (46), which explains why bats are asymptomatic virus hosts. That is to say, the ability of batMDA5 to activate IFNβ after virus invasion is relatively mild, which was also consistent with previous studies that virus or ploy(I:C) stimulation did not induce the activate IFNα. Therefore, we believe that bat MDA5 can sense virus invasion and activate IFNβ, but this activation is relatively mild, which is one of the reasons why bats can colonize hundreds of viruses without the disease.

## Data Availability Statement

The original contributions presented in the study are included in the article/**Supplementary Material**. Further inquiries can be directed to the corresponding authors.

## Author Contributions

JW, YC, and JS designed the research and analyzed the data. JW, ZL, QL, FF, ZW, JM, HW, YY, and JS conducted the experiments and collected the data. JW and YC wrote the paper. All authors approved the final version of the manuscript.

## Funding

This research was supported by the National Natural Science Foundation of China (32072864 and 32072865), the Natural Science Foundation of Shanghai (20ZR1425100), Science and Technology Commission of Shanghai Municipality (21N41900100), Shanghai Agriculture Applied Technology Development Program, China (2022-02-08-00-12-F01191).

## Conflict of Interest

The authors declare that the research was conducted in the absence of any commercial or financial relationships that could be construed as a potential conflict of interest.

## Publisher’s Note

All claims expressed in this article are solely those of the authors and do not necessarily represent those of their affiliated organizations, or those of the publisher, the editors and the reviewers. Any product that may be evaluated in this article, or claim that may be made by its manufacturer, is not guaranteed or endorsed by the publisher.
