# Supplementary material for: Bat Employs a Conserved MDA5 Gene to Trigger Antiviral Innate Immune Responses
Source: Front Immunol. 2022 May 23;13:904481. doi: 10.3389/fimmu.2022.904481 (PMC9168228; doi:10.3389/fimmu.2022.904481)
Supplement: Supplementary file 1 [file DataSheet_1.pdf]

Supplementary Material

1 Supplementary Table 1 Primer sequences

Genes	Forward Primer (5'-3')	Reverse Primer (5'-3')
batMDA5	ATGACGAACGGGCATTCTGCA	TCAATCTTCATCACTAAACAA
batMDA5-Flag	AGTGTGGTGGAATTCATGACGAACGGGCAT	CTTGTAGTCCTCGAGATCTTCATCACTAAA
batMDA5-C1-Flag	ACGAACGGGCATTCTTCCTTTGAGAACAATCAT	AGAATGCCCGTTCGTCAT
batMDA5-C2-Flag	ATCCTTTGAGAACAAGCCTCTGACAGCAGCGCA	TTGTTCTCAAAGGATGGA
batMDA5-ATP-Flag	AGTATTCCCTGAGCCGAGAGCACCTACGTCCTG	GGCTCAGGGAATACTCTTTCT
batMDA5-CTD-Flag	AAACCCATCATTAATTGTTTGTTTAGTGATGAA	ATTAATGATGGGTTTTCTTTA
batMDA5-CARD-Flag	CTCGAGGACTACAAGGAC	CTTGTAGTCCTCGAGGTTGGAGCCTGTCAA
qbatMDA5	TTCAGCCCACTCTGGTGGACA	TCTCTTTCTGCACAATCCTTC
qbatIFNβ	GCACCGGCTGGAATGAGACCA	GTCCAGGCATTGGCTGT
qbatMX1	GGAGGGTCAGCTCCCCTCA	GCCATGCTCAGCGCCTCT
qbatOAS1	ATCTGCAGTTTCCTGAAGGAG	GCTGAGGAAGCGACGAGGTC
qbatIL-6	CTACTGCTTTCCCTACCC	TCCTTGCTGTTTTTACACG
qbat β-actin	CCATCCTGCGTCTGGACCTGG	GTGGCCATCTCCTGCTCGAAG

Supplementary Figures

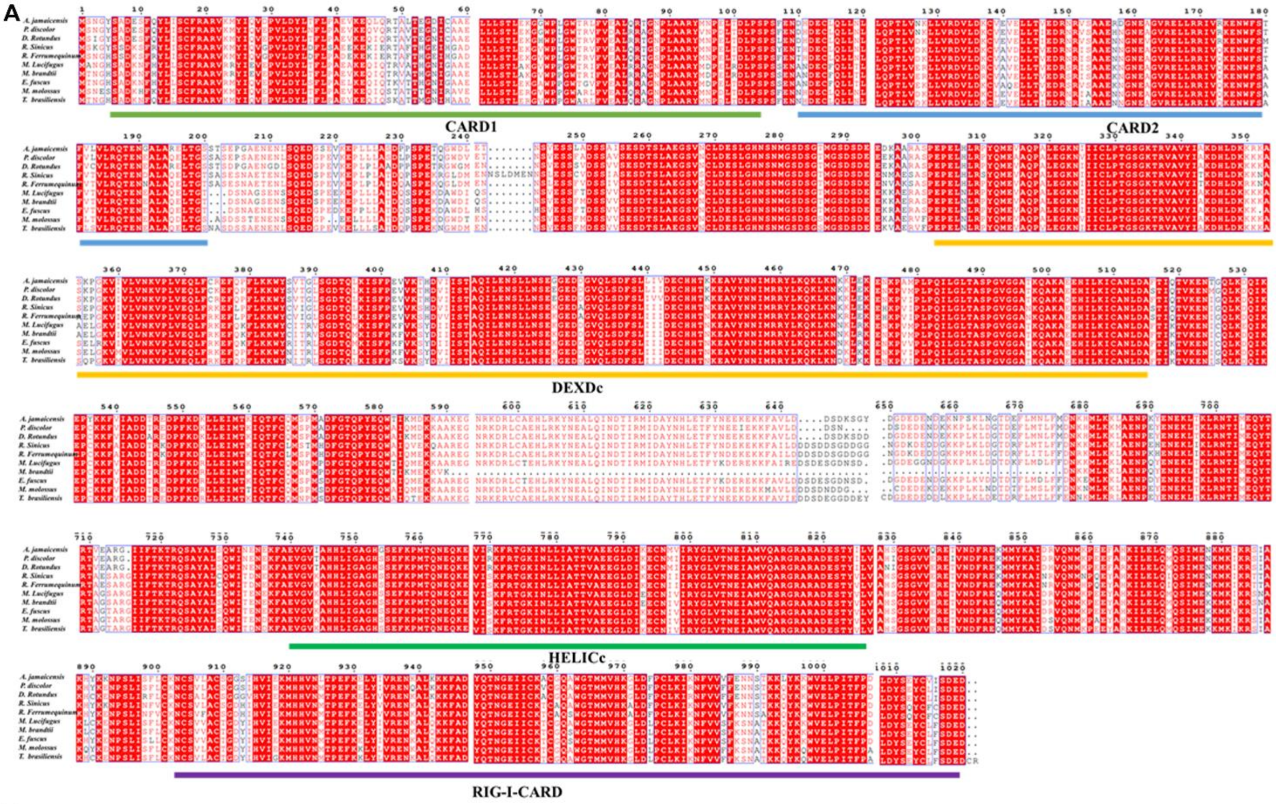

B

Percent Identity (upper triangle); Divergence (lower triangle)

	1	2	3	4	5	6	7	8	9	10		
1		21.5	21.6	56.3	55.2	25.4	56.9	56.3	23.8	21.3	1	*T. brasiliensis*
2	230.0		36.5	26.6	26.3	19.4	21.6	20.9	56.2	59.7	2	*M. Lucifugus*
3	229.0	128.5		23.3	47.4	20.2	22.7	21.7	33.4	36.3	3	*E. Fuscus*
4	56.4	177.3	206.0		67.6	25.2	61.8	56.6	29.3	27.0	4	*D. Rotundus*
5	66.3	193.9	88.9	35.9		25.6	62.5	55.6	23.4	21.0	5	*A. Jamaicensis*
6	193.9	252.0	241.0	180.5	190.4		24.9	29.6	20.5	18.4	6	*R. Ferrumequinum*
7	62.1	236.0	224.0	47.3	52.0	195.7		56.8	23.0	22.0	7	*P. discolor*
8	64.5	240.0	231.0	56.4	66.1	161.5	62.9		21.6	19.9	8	*R. Sinicus*
9	196.0	57.8	133.8	203.0	203.0	221.0	212.0	217.0		58.0	9	*M. brandtii*
10	211.0	47.1	115.5	220.0	223.0	241.0	216.0	231.0	64.4		10	*M. molossus*
	1	2	3	4	5	6	7	8	9	10		

Supplementary Figure 1. Conservation analysis of MDA5 in different species of bats. (A) Alignment of the deduced amino acid sequence of Tadarida brasiliensis MDA5 with other bats species MDA5 proteins. This was performed using the Clustal X and edited with ESPript 3.0. (B) The amino acid sequence homology of different animals.
